# Assessing the Feasibility and Acceptability of a Virtual Food Skills and Food Sustainability Program Designed for Children Living With Type 1 Diabetes

**DOI:** 10.1155/2024/3821265

**Published:** 2024-10-22

**Authors:** Sarah Goldstein, Olivia Chow, Joeie Schwartz, Vanita Pais, Susan Wright, Enza Gucciardi

**Affiliations:** ^1^School of Nutrition, Toronto Metropolitan University, M5B 2K3, Toronto, Canada; ^2^summerlunch+, Toronto, Canada; ^3^The Hospital for Sick Children (SickKids), M5G 1X8, Toronto, Canada

## Abstract

**Objective:** To assess the feasibility and acceptability of a virtual food skills program for children with type 1 diabetes.

**Methods:** Forty-three patients, aged 6–14 years with type 1 diabetes, participated in an 8-week online programme, summerlunch+ At Home, that included weekly live cooking classes, asynchronous learning modules, and quizzes accessed through Google Classroom. Grocery delivery or gift cards were provided to all participants to support equitable access to participation. Descriptive results were summarized, and thematic analysis was performed on answers to a post-intervention questionnaire, parent/caregivers interview transcripts, and facilitators' field notes.

**Results:** Participants reported having a positive experience and would recommend the programme to others. Acceptable elements included the online format, the cooking class demonstrations, and the well-organized content. Families enjoyed the recipes, expressed an improvement in the families' cooking skills and nutrition knowledge, and noted the program as a way to improve family bonding and reduce participants' sense of social isolation given the opportunity of meeting peers with diabetes. The intervention also appears to increase participants' independence, confidence, and self-esteem. While grocery cards were easier to coordinate compared with meal kits, both were deemed acceptable by caregivers. Barriers to participation include a distracting home environment and not feeling comfortable on camera. Factors that negatively impacted satisfaction were the large age range of participants and the class timing and duration. Caregivers noted a desire for more diabetes education, enhanced peer-to-peer interaction, and incorporation of animal-based protein recipes in future programmes.

**Conclusion:** The current study demonstrates the feasibility and acceptability of the virtual summerlunch+ At Home cooking and nutrition program that was adapted for children with diabetes. Similar food skills programmes may support the development of food skills imperative to diabetes self-management long-term. Further research can continue to assess food literacy skills, glycemic management, and the social benefits of such interventions.

## 1. Introduction

More than 500,000 children under 15 live with type 1 diabetes worldwide, with Canada ranking 8th amongst countries with the highest incidence [1]. Dietary management is integral to diabetes management in children with type 1 diabetes. It is critical in achieving optimal glycemic control, preventing future micro- and macrovascular complications and promoting general long-term health [2–4]. However, the current complex food environment makes it difficult for children to navigate and make nutritious choices. Additionally, society's reliance on convenience foods has resulted in the “de-skilling” of domestic cooks [5].

Studies among general paediatric populations find that educational programmes that encompass most or all of the food literacy competencies, including the understanding, accessing, appraising, and applying of health and food-related information [6], are associated with improvement in food choices and attitudes towards healthy or new foods, increased consumption of healthy foods (e.g., fruits and vegetables), and self-efficacy in cooking and food preparation [7, 8]. However, associated changes in health indicators such as BMI and measures of overall well-being results are mixed [7]. Studies also show that the paediatric populations report high enjoyment from educational programmes and could reap nonfood-related benefits such as improved social skills, cultural appreciation, and increased family and community engagement while participating in selected interventions [8].

Most of the literature on food literacy interventions has been conducted in in-person settings [7, 8]. Since the COVID-19 pandemic, some in-person self-management interventions targeting adults with conditions such as type 2 diabetes and osteoarthritis have transitioned to virtual programming. These virtual programmes demonstrate increased convenience and accessibility while continuing to deliver intended positive outcomes (e.g., increase disease management knowledge and weight loss) for participants [9, 10]. Moreover, recent studies on virtual food literacy and cooking education for adults without diabetes have shown preliminary success in increasing fruit and vegetable consumption, enhancing self-efficacy in healthy eating, and reducing BMI [11, 12]. A cooking-based intervention that targeted adults living with type 1 or type 2 diabetes demonstrated improvement in participants' diet, including the reduction of refined grain and sugar consumption [13].

In summary, although cooking-based interventions have been completed with positive outcomes via in-person channels for general paediatric populations and adults with diabetes and via virtual channels with adults with and without diabetes, currently, no existing research was found on food literacy programmes, either in-person or virtual, for the paediatric population living with type 1 diabetes. The current intervention will be able to address this gap in the literature, as we aim to assess the feasibility and acceptability of implementing summerlunch+At Home (https://www.summerlunchplus.com/), an 8-week comprehensive virtual food skills and food literacy education programme tailored for children with type 1 diabetes. To gain a well-rounded perspective, this study focuses on the self-reported experiences of child participants and their families with the programme, as well as programme facilitators' observations and experiences on implementing the summerlunch+ At Home programme.

## 2. Methods

This study was approved by both the Toronto Metropolitan University and the Hospital for Sick Children Research Ethics Board. Parents provided consent for themselves and their children before starting the programme and completing the baseline questionnaire.

### 2.1. Programme Intervention

The summerlunch+ programme is a well-established Toronto-based food skill and food literacy education programme that teaches children in priority neighbourhoods about cooking, nutrition, healthy eating for wellness, and environmental sustainability. In response to the COVID-19 pandemic, an 8-week virtual format called summerlunch+ At Home was developed and implemented in 2020–2023. The summerlunch+ At Home programme was based upon a comprehensive assessment of the literature and Food Literacy Framework by the Locally Driven Collaborative Project (LDCP) Healthy Eating Team [14]. For the current study, the summerlunch+ At Home programme has been adapted for children with type 1 diabetes by integrating nutrition education from the 2018 Clinical Practice Guidelines [15], such as healthy eating guidelines and glycemic index of foods.

The classes were facilitated by a dietetic intern and a nutrition student, with class content reviewed by a registered dietitian (RD). The focus was food skills and food literacy. Children participated in the programme through Google Classroom, where learning modules were released every Monday, along with three vegetarian and halal recipes. Recipes were vegetarian to promote increased consumption of plant-based proteins and sustainability. Providing vegetarian recipes supports Canada's Food Guidelines recommendation to incorporate plant-based protein daily [16]. Additionally, vegetarian recipes tend to be more economical for families, created logistical ease for meal kit delivery, and minimized food safety concerns. The content of the programme and all recipes were reviewed by a Certified Diabetes Educator (CDE) and RD, and all recipes listed the nutrition content, with meals containing 40–50 g of carbohydrates and snacks containing 15–20 g.

**Figure 1:**
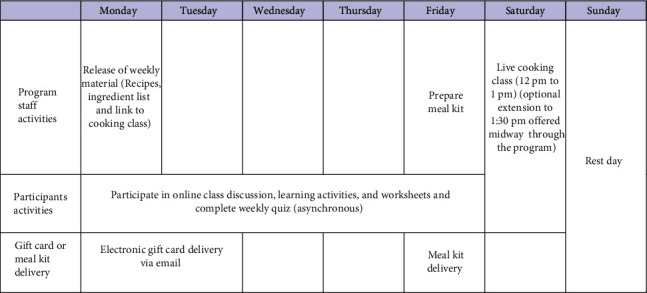
Weekly schedule during the intervention.

Additionally, facilitators used the Google Classroom to provide programme updates, engage with participants, and share Zoom links for the cooking classes. Participants signed up with their personal Gmail addresses or were provided with a unique email if they did not have an account or if they wanted to remain anonymous for the duration of the programme. Each week, participants were instructed to review a module and then complete a quiz. Participants also had opportunities to participate in online class discussions and learning activities and worksheets On Saturday afternoons at 12:00 pm, and summerlunch+ staff conducted live cooking classes on Zoom beginning with a review of the asynchronous module content following with the cooking activity. (Refer to Figure 1 for weekly schedule.) To monitor participation and create a more engaging environment, children were asked to turn on their cameras. If they decided to leave their cameras off, they were instructed to post a photo of their prepared meals following the class.

Please refer to File S1 for weekly lesson plans.

### 2.2. Meal Kit Delivery and Gift Cards

To enable access and equitable participation in the programme, all participants were offered either a free meal kit delivery that included all necessary ingredients or a $30.00 grocery card weekly. Meal kits were procured and packaged by the summerlunch+ staff at their facility, and delivery was provided for those living within the Greater Toronto Area (GTA). Participants received the meal kits on Friday afternoon before the Saturday cooking class. Families who lived outside of the GTA and those with specific allergies or religious requirements received electronic grocery gift cards at the beginning of each week via email, along with a shopping list. If participants missed three consecutive cooking classes, meal kit delivery/electronic grocery cards were stopped unless they informed us of their absence.

### 2.3. Study Design

Immediately post-intervention, a questionnaire with four open-ended questions was administered to participants, and within 1 month after the intervention, interviews were conducted with 10 parents/caregivers of children who participated in the intervention to discuss the acceptability and feasibility of the intervention. Although participants also completed questionnaires with close-ended questions at 3 months and 6 months following completion of the intervention, these data were not analyzed for this paper.

### 2.4. Recruitment and Study Participants

Participants were recruited through the Hospital for Sick Children in Toronto, Canada. An email was sent to all eligible participants between the ages of 6 and 12 and spoke English. Exclusion criteria included a diagnosis of diabetes less than 1 year and did not have access to a computer or an internet-connected device. Healthcare providers within the diabetes clinic also introduced the intervention to their patients. Interested parents/caregivers/patients were instructed to sign up and subsequently enrolled in the study by the research assistant. Participants were paid $10.00 to complete each questionnaire and $20.00 for an in-depth interview.

### 2.5. Assessment

#### 2.5.1. Questionnaire

The pre-intervention questionnaire collected demographic information and assessed baseline food security. The immediate post-intervention questionnaire included four short answer questions related to the acceptability and feasibility of the intervention program, which were analyzed in this manuscript. The four questions were as follows: (1) describe your experience of accessing the summerlunch+ At Home programme via the internet/online; (2) what prevented you from making all of the weekly recipes; (3) please list two things you liked about the summerlunch+ At Home programme; (4) please list two suggestions for change; and (5) do you have any comments that would help us improve the programme.

#### 2.5.2. Interviews

An email was sent to all the programme participants' parents/caregivers asking if they would like to participate in one-on-one interviews and be asked questions about their experience of taking part in the programme, 10 individuals volunteered, and all were interviewed. An interview guide was developed and used during the interviews. Clarifying questions and prompts were used throughout the interview to ensure the interviewer's adequate understanding. Meeting links were sent through email, and interviews were conducted on Zoom, with optional video.

The interviews were facilitated by one research team member who received verbal consent to record audio from the interview. A second research team member transcribed the interviews.

Please refer to File S2 for the interview guide.

#### 2.5.3. Field Notes

Field notes were also completed by two intervention facilitators and one research team member throughout the duration of the 8-week programme. Immediately after each cooking session, the participating team members would record their reflections and observations regarding the delivery of the online cooking class. These included weekly feedback from parents/caregivers or participants, technology concerns on the online platform, issues with general operations, and challenges with the grocery cards or meal delivery which were noted. Notes were compiled, synthesized, and included for data analysis.

### 2.6. Data Collection and Management

The surveys were created, administered, and stored through REDCap, a secure web-based data management platform. The audio from the interviews was recorded and stored on a password-protected computer and within a password-protected folder. The audio was transcribed by a research assistant with personal identifiers removed. The field notes did not include names or any personal identifying information for any participants of the programme or of the research team members.

### 2.7. Data Analysis

We took an inductive analytical approach to the qualitative coding process [17] and conducted specific steps for the analysis of data [18]. Analysis was performed on short answer questions, interviews, and field notes. Three research members familiarized themselves with the text data and independently identified specific text segments as themes that relate to the evaluations of the acceptability and feasibility of the intervention. Codes were then categorized into independently constructed categories. When combining each team member's work, overlap and redundancy were removed, and the team agreed on the structure of the codes and themes. All research team members completed a series of revisions and refinement of the combined category system until a concise list of themes emerged. Descriptive results summarized the demographic data and were reported as means and standard deviations.

## 3. Results

The baseline questionnaire was filled out by 63 participants. The baseline demographic and clinical information at the time of enrolment can be found in Table 1. Of the participating children, 42.8% identified as female, and most children were in grade seven or higher (52.4%), followed by grade four to six (38%), then grade three, and under (9.5%). The mean HbA1c was 7.8% (SD ± 1.4), the mean age of diagnosis was 6.6 (SD ± 3.3). The largest group of participants (35.5%) self-identified as “White/Caucasian”, followed by “Black/African Canadian” (22.6%). Overall, 17.5% (*n* = 11) of families reported being food insecure, based on answering “occasionally,” “almost always,” or “always” to any of the three following questions (adapted from the US Household Food Security Survey [19]) using the past 6 months as time reference: “Did you ever worry whether your food would run out before you got money to buy more?”; “Was there ever a time when the food you bought just didn't last, and you didn't have money to get more?”; and “Did you or others in your household cut the size of your meals or skip meals because there wasn't enough money for food?”

Of these initial participants, 43 children completed at least five out of the eight cooking sessions. By the end of the programme, 19 participants received weekly grocery cards, and 24 received food kits.

The immediate post-intervention questionnaire was filled out by 46 participants, of which 45.6% were parents, 41.3% by parents and children, and 13.0% by the children themselves. The children who filled out the questionnaires themselves were between the ages of 7 and 13 years. The mean age of participants was 10.5 years with standard deviation ±2.35. In depth post-intervention interviews were conducted with 10 volunteers. The research team considered a sample size of 10 interviews sufficient as data saturation was reached when similar themes consistently emerged from the interviews.

**Figure 2:**
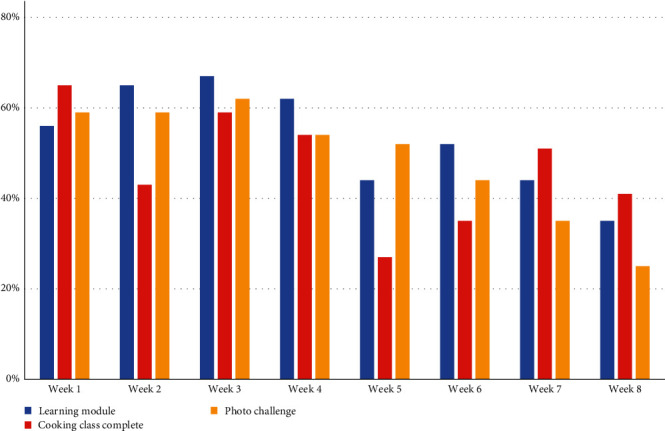
Weekly completion rates of the intervention components. *Note:* The number of participants who have completed the baseline questionnaire (63) is used as the denominator in the calculation of the participation rate.

Participation rate in the asynchronous modules, the live cooking classes, and the photo challenges all slightly decreased as the intervention progressed (Figure 2). The drop in live participation in Week 5 coincided with a public holiday.

### 3.1. Result of Thematic Analysis

**Figure 3:**
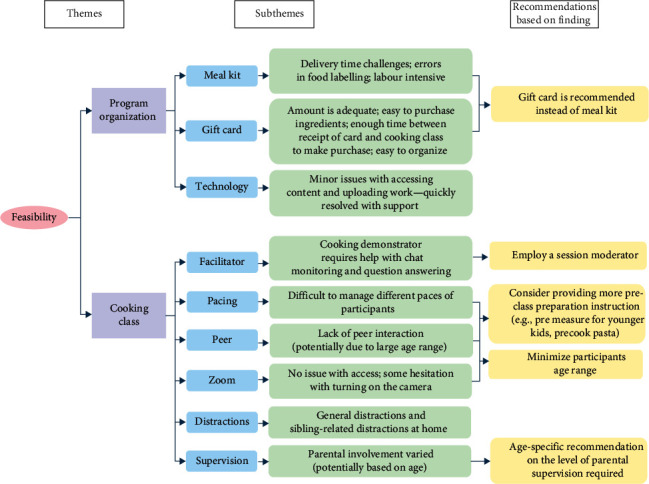
Summary of feasibility-related themes and corresponding recommendations.

**Figure 4:**
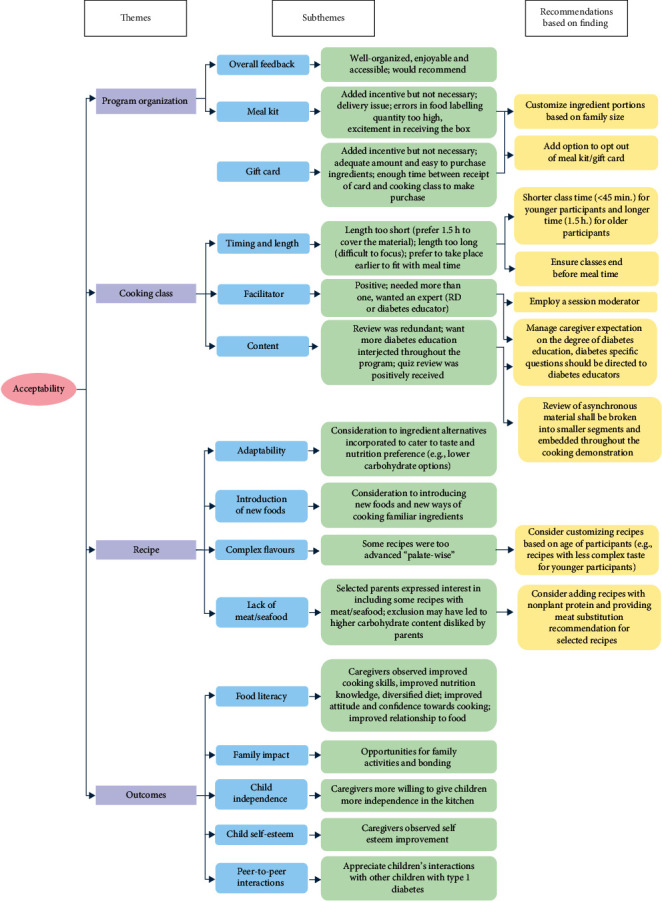
Summary of acceptability-related themes and corresponding recommendations.

Analysis of feasibility is outlined in Figure 3 with two main themes: programme organization (subthemes: meal kit, gift card, and technology) and cooking classes (subthemes: facilitator, pacing, peer, zoom, distractions, and supervision). Analysis of acceptability is outlined in Figure 4 with four main themes: programme organization (subthemes: overall feedback, meal kit, and gift card), cooking class (subthemes: timing and length, facilitator, and content), recipe (subthemes: adaptability, introduction of new foods, complex flavours, and lack of meat/seafood), and outcomes (subthemes: food literacy, family impact, child independence, child self-esteem, and peer-to-peer interactions).

### 3.2. Feasibility

#### 3.2.1. Theme 1: Program Organization

Both participants who received meal kits and those who received grocery cards were successfully able to participate in the intervention. Purchasing and organizing food, accurately labelling foods, and coordinating the delivery of the meal kits were very labour-intensive. In contrast, grocery cards were easy to purchase and distribute, and participants reported ease in procuring ingredients given the monetary amount and time provided. No major issues were identified with the technologies used (i.e., Google Classroom, Zoom, and email) throughout the intervention. There were minor initial issues with accessing content (i.e., inability to sign in to Google Classroom) and uploading the completed work (i.e., quiz and pictures of completed dishes). However, issues were quickly resolved by the administrative team.

#### 3.2.2. Theme 2: Cooking Class

The intervention began with only the cooking class facilitator, which proved to be challenging in maintaining control and engagement of the class. In the third week, a session moderator was added to monitor the chat box and answer participants' questions, resulting in the class running more effectively.

Participation levels varied by age, as was participants' ability to follow instructions, which affected class flow. Older participants were hesitant to turn on their cameras in Zoom, limiting their engagement with others. Younger participants were easily distracted by other siblings or home events. Many parents also reported that the participants' age range was too large, which may have impacted engagement. One parent mentioned, “the age difference was a hurdle for her [the child participant]. Just cause the pace”. Some parents also mentioned that although there were interactions between the facilitator and the participants, they wish there could be more peer-to-peer interactions. Lastly, parental involvement in the intervention varied greatly. Some leave their child to participate independently, while others supervised their child throughout the class and assisted with recipe preparation.

### 3.3. Acceptability

#### 3.3.1. Theme 1: Program Organization

The overall feedback on the intervention was highly positive; it was reported as well-organized, enjoyable, and both physically and technologically accessible, with many expressing that they would recommend the intervention to others. Both the meal kits and grocery cards were highly acceptable. Participants shared that although the meal kits and grocery cards served as an incentive, they would still participate in the programme had they not been offered. For the meal kits, some participants expressed that there were delivery issues, that some ingredients were not labelled (i.e., spices), that the quantity of ingredients was too much for smaller families, or that the ingredients provided were already stocked at home or not fully utilized by the families which led to food waste. However, many parents also shared that their children anticipated receiving the kits each week and were excited to unpack the ingredients.

#### 3.3.2. Theme 2: Cooking Class

The 1-h class each week was reported to be too short to cover all the materials and cook two full recipes. As a result, the second half of the intervention classes was lengthened to 1.5 h. However, some participants felt classes were too long, primarily for younger participants who lost focus towards the end of the session. Some parents voiced concern that the timing of the classes, which took place from 12 pm to 1:30 pm on Saturdays, passed participants' mealtime, disrupting eating schedules and impacting their child's blood glucose management.

The cooking demonstration was received positively by families. The intervention focus was on food skills and food literacy; however, multiple caregivers voiced a preference for additional diabetes content and a CDE or person with lived diabetes experience fielding diabetes-related questions. (These participants were advised to contact a member of their personal care team to discuss any questions related to their personal diabetes management.)

Additional feedback included removing the weekly module content review at the start of class due to redundancy for those who watched it in advance. Participants preferred the review in the form of a quiz that took place at the end of class.

#### 3.3.3. Theme 3: Recipes

Most families enjoyed the recipes as they introduced new foods and ways of cooking. One caregiver reported there was “an interest in trying some new things… and mixing ingredients together in a way that [the child] wasn't always used to”. And another caregiver expressed that the child “was trying food that she would never have tried before”. Many appreciated ingredient substitutes provided in recipes to cater to the taste and nutrition preferences (e.g., lower carbohydrate options) of their children. However, some caregivers expressed that the flavours of some recipes were too complex for their child, and some preferred the inclusion of meat or seafood recipes and recipes with lower carbohydrate content.

#### 3.3.4. Theme 4: Outcomes

Caregivers reported an increase in their childrens' food literacy. They observed improved cooking skills and nutrition knowledge and a greater diversification of their family diets. One caregiver mentioned that her child “knows how to read the nutrition label now and what it means”, another reported that their child is “making better choices in terms of foods that don't spike blood sugars… watching ingredients and picking ingredients that maybe are more natural than artificial”. It was also noted that there was an improved attitude and confidence towards cooking as one caregiver reported, the child “was more into cooking and wanted to cook more”. Lastly, there was also an improved relationship to food as one caregiver noted that the intervention has “built [the child's] foundation about good food habits”.

The intervention provided opportunities for family activities and family bonding. One caregiver recited what her child had expressed “it's nice to be just in the kitchen and doing things without rushing off to go somewhere”, and that her child enjoyed having that one-on-one time each week.

The intervention appeared to increase some children's independence, confidence, and self-esteem. The intervention also helped caregivers become more comfortable passing on some diabetes management responsibilities and cooking-related activities to their child and “to just have confidence in [the child] and know that [the child] can reach out for support” when needed. Another caregiver also mentioned that they are “calmer now when [the child] is in the kitchen”.

Lastly, caregivers noted that the intervention created an opportunity for their children to interact with other children with type 1 diabetes. One caregiver mentioned, “it was the first event where my [child] got a chance to meet other kids with type 1 diabetes… in a group setting.” Another caregiver described the experience “having her exposed to other people, even if it's virtual, has been good for her, because since she was diagnosed, she's [been] isolated. I know the pandemic has not helped out, but she's isolated herself a little bit. Because eating out with friends is harder, explaining why she has to do what she does is harder, especially at her age. So, I think seeing all those other kids in the class and how some of them were so outgoing and happy, willing to share their experiences was beneficial to [the child] as well.”

## 4. Discussion

These pilot study results demonstrate the feasibility and acceptability of a virtual food literacy programme for paediatric type 1 diabetes patients. The provided meal kits and grocery cards facilitated engagement. Providing food kits or grocery cards may also increase uptake from those most vulnerable, such as families who are food insecure. The virtual technology used was generally easy to handle, consistent with increased virtual learning comfortability post-COVID-19 [20, 21]. The facilitation and technology provided sufficient support for participants to complete the intervention, similar to other virtual food literacy interventions for adults [11, 12].

The intervention, including programme organization, cooking classes, and recipes, received positive feedback from participants and caregivers. The reported outcomes align with in-person food literacy studies on the general paediatric populations, showing improved food literacy, cooking skills, nutrition knowledge, diet diversity, attitude, confidence, and a more positive relationship with food [7, 8]. It also fostered increased self-esteem and community cohesion through peer interaction, like other in-person food literacy studies for children and youth [8].

The virtual and at-home nature of the intervention fostered family bonding, as caregivers worked with their children on tasks together, and older participants shared their experiences and creations during family meals. Future virtual programmes should leverage the unique characteristic of at-home participation and incorporate activities that can promote family involvement and interaction. These enhancements can strengthen family relationships, which are also positively associated with improved diabetes self-management [22]. The repetitive at-home nature of the intervention may foster healthy cooking habits in children's home environment [23]. The potential of installing healthy cooking habits in children is worth exploring.

Some parents expressed that the intervention allowed them to comfortably promote more independence regarding cooking activities and managing their diet. The increased autonomy in food selection and preparation may enhance children's self-efficacy and better prepare them for dietary and diabetes management as they age. Moreover, it is common for parents to experience high levels of stress and be in a constant state of vigilance when managing a child with type 1 diabetes [24, 25]. Parents may also struggle with transitioning care autonomy to their children as they age due to their perception of poor food skills relating to disease management [26]. Thus, this intervention has the potential to relieve paediatric parenting stress as children become more knowledgeable about healthy eating and efficacious in food skills.

Like other food literacy studies, paediatric participants enjoyed peer-to-peer interactions in group programmes. The virtual element of this intervention facilitated a connection with children who shared experiences from across the GTA in an accessible and safe manner.

For children with type 1 diabetes, this aspect is particularly crucial due to potential feelings of isolation related to their condition. Interacting with other children in a nonclinical setting that focuses on increasing food literacy versus disease management may lessen their sense of loneliness and social isolation and positively impact their attitude towards diabetes management.

### 4.1. Recommendations

In the pilot study, various ways to improve the intervention were identified by the researchers based on thematic analysis (Figures 3 and 4, highlighted in yellow). Meal kits and grocery cards were effective incentives, but many parents said they would still enrol without them. Some preferred receiving smaller portions or purchasing their own ingredients to accommodate smaller families or avoid duplication of ingredients and reduce waste. For future programmes, customizing the incentives based on family size is recommended. Offering an opt-out for these incentives can better allocate resources to families facing financial barriers and increase acceptability for families with sufficient means. Considering programme implementation, grocery cards are preferable to meal kit delivery, as they are less labour-intensive and equally accessible and acceptable. This is especially relevant in urban centres where participants regularly access grocery stores. Additionally, the use of grocery cards can also resolve the portion size mismatch and food wastage issue mentioned above.

It is recommended to narrow the age range of participants, allowing for better customization. For younger participants, class length should be shortened to accommodate their limited attention span, while older participants can have longer classes to enhance engagement and knowledge transfer [27]. Caregiver involvement is highly recommended for younger participants to assist with measuring, cutting, and pre-cooking ingredients, while older participants can complete the classes more independently. Additionally, recipes for younger participants should be simpler to increase acceptability, while recipes for older participants can be more complex to enhance food literacy and dietary variety.

Due to the low acceptance from parents of the review of asynchronous module content at the beginning of live class, the review content shall be broken into smaller segments and be embedded throughout the cooking demonstrations. We also suggest maintaining the addition of a session moderator, who fields questions, in addition to the cooking facilitator who leads the sessions. To enhance the intervention specifically for participants with type 1 diabetes, some specific considerations should be considered:

Aligning live classes with regular mealtimes: Participants with diabetes typically adhere to regular meal schedules for blood glucose monitoring. Thus, it's important to schedule live classes before mealtimes to allow participants to consume their cooked dishes on time.

Managing caregiver expectations on the degree of diabetes education: In addition to the programme content being reviewed by an RD and facilitated by dietetic students, some caregivers may expect more diabetes-focused education directly from CDEs. As the intervention aims to promote a focus on food literacy rather than diabetes education, it is essential to address these expectations beforehand. Programme administrators should clarify that diabetes-specific questions, especially those specific to individual patients' care, should be directed to patients' healthcare team, emphasizing the intervention's focus on food literacy to support diabetes management.

Addressing carbohydrate concerns: While the recipes provided followed the Canadian Diabetes Guidelines and were reviewed by an RD, some caregivers expressed concern about the high-carbohydrate content and requested the addition of lower carbohydrate foods such as meat- or fish-based recipes. Families are encouraged to adjust and adapt recipes to meet their needs, and efforts can be made to provide meat or fish alternatives for all recipes. The programme can also be an opportunity to discuss the benefit of including plant-based protein sources for children with diabetes as an economical way to provide essential nutrients and fibre to support blood sugar management.

### 4.2. Limitations

The exclusion of participants who could not speak English or did not have access to a computer or internet connecting device may lead to the exclusion of lower income, newcomer, and food insecure households that may potentially benefit from the food literacy intervention. Additionally, recruiting participants from lower income households was also challenging as the caregivers may be dealing with multiple employments leading to an inability to participate alongside their child due to time restraints.

The virtual nature of the programme and participants' ability to turn off their cameras (primarily among the older participants) made direct observation of active participation challenging. Therefore, other forms of participation was recorded such as completion of the learning modules and submission of the photo challenge. Most of the reporting was done by parents instead of the participants, which may reflect the caregivers' perspective instead of the participants' experience. The at-home environment, such as the level of access to technology, parental guidance, the amount of engagement, or distraction posed by siblings can potentially impact the participant's experience but was not accounted for in the current study.

## 5. Conclusion

In conclusion, the current pilot study demonstrated the feasibility and acceptability of a virtually delivered food literacy intervention tailored for children with type 1 diabetes. The positive outcomes in food literacy and other social aspects align with previous in-person interventions. Moreover, the targeted approach to this specific population yields additional social benefits, such as reduced caregiver stress, increased participant self-efficacy, and enhanced community cohesion. Food skills and literacy interventions must consider the nuanced needs of children with diabetes, including class timing according to mealtimes and low-carbohydrate recipes. Future studies with larger sample sizes and longer follw-up periods can further validate and build upon these promising preliminary results. Ultimately, food literacy interventions can bring significant benefits to the type 1 diabetes community by improving dietary management, social well-being, and overall quality of life for children and their caregivers. Furthermore, delivering a virtual intervention reduces costs and increases accessibility for participants eliminating geographical barriers.

## Figures and Tables

**Table 1:** Descriptives of the demographic information at baseline (total number of participants *n* = 63).

Participant characteristics	Mean	Stdev
HbA1c (%)	7.8	1.4
Age of diagnosis	6.6	3.3

	**Number of participants**	**Percent (%)**

Grade		
≤Grade 3	6	9.5
Grade 4 to Grade 6	24	38
≥Grade 7	33	52.4
Gender		
Female	27	42.8
Male	36	57.2
Ethnicity		
Caucasian	22	35.5
African Canadian	14	22.6
Asian	4	6.4
Southeast Asian	4	6.4
Hispanic/Latino	1	1.6
Indigenous Canadian	1	1.6
Other	14	22.6
Prefer not to answer	2	3.2
Food security in past 6 months		
Secure	52	82.5
Not secure	11	17.5

## Data Availability

The data that support the findings of this study are available from the corresponding author, Enza Gucciardi, egucciar@torontomu.ca, upon reasonable request.

## Supporting Information

Supporting Information File S1: summerlunch+ weekly learning objectives and diabetes lessons outline. File S2: Caregiver interview guide.
