# Supplementary material for: Assessing the Feasibility and Acceptability of a Virtual Food Skills and Food Sustainability Program Designed for Children Living With Type 1 Diabetes
Source: Pediatr Diabetes. 2024 Oct 22;2024:3821265. doi: 10.1155/2024/3821265 (PMC12016876; doi:10.1155/2024/3821265)
Supplement: Supporting Information — File S1: summerlunch+ weekly learning objectives and diabetes lessons outline. File S2: Caregiver interview guide. [file 3821265.f1.pdf]

Supplement 1

Overview of Summerlunch+ Program with Adaptations with SickKids

Week 1: Kitchen and Food Safety

Learning objectives:

- Familiarize children with their home kitchen environment
- Practice kitchen and food safety (i.e., hand hygiene, when to ask for help, cleaning, proper storage of food)
- Develop safe knife handling and skills
- Practice proper measurements of ingredients

Diabetes Lessons:

General kitchen safety and basic food skills in knife handling and measurements are essential to food literacy for all children. Learning measurements commonly used in cooking such as cup or tablespoon measures, familiarizes children with diabetes with portion sizes. Food measurements are important to diabetes management and an understanding of them helps better estimate portion sizing of meals and snacks.

Week 2: Seed to Table

Learning objectives:

- Familiarize children with food production practices and the resources involved in getting food to the table
- Acknowledge the importance of using local and in-season produce when possible
- Apply critical thinking about the distance that food has to travel and how to move towards sustainable options

Diabetes Lessons:

An understanding of where food comes from and the resources required to produce food is a universal concept for all children as it improves food knowledge, informs food choices, and increases self-efficacy to make healthier choices for oneself and the environment. However, it is especially important for children with diabetes to consume a diet with fresher and less processed foods. By learning the processes involved in getting food to the consumer, children can gain a better appreciation of food from its source and use that knowledge to make healthier choices for themselves.

Week 3: Balanced Meals

Learning objectives:
- Understand the importance of consuming balanced meals and the different food groups
- Practice using the Canada Food Guide's plate model to build balanced meals
- Acknowledge the role of fibre on the body and how it plays a role in diabetes, and provide examples on sources of fibre
- Differentiate between the types of fats and its effect on the body

Diabetes Lessons:

Consuming a balanced meal and diet is crucial to diabetes management. Diabetes Canada Clinical Practice Guidelines state that children with diabetes should follow a healthy balanced meal, the same as those without diabetes, however, there are additional nutrients to be cognizant of in diabetes management. This includes understanding the role of carbohydrates and the effect on blood glucose. The module will reinforce the teachings of the SickKids Diabetes Clinic on balancing carbohydrates, fats and proteins, and highlight carbohydrate and carbohydrate-free sources. Additionally, the module will inform children on the role of fibre in diabetes management. The recipes provided by summerlunch+ will have the nutritional content posted for participants to review to help ensure that their meals are balanced.

Week 4: Physical activity and hydration

Learning objectives:
- Understand the role of physical activity and health
- Develop a plan to include physical activity into daily routines
- Recognize the role of having proper fuel for exercise

Diabetes Lessons:

Adequate and nutritionally appropriate fuel for exercise is important for all children, however, children with diabetes need to take extra care when preparing to participate in physical activity. The module will reinforce the precautions taken to exercise safely to prevent hypoglycemic events. As children often consume sugar-sweetened beverages during exercise, such as Gatorade, the module will remind children that these beverages cause a blood sugar spike and to be mindful when consuming them.

Week 5: Food Labels and Marketing

Learning objectives:
- Accurately locate Nutrition Facts table on food packages and understand their role
- Identify key nutrients such as sugar, fat, and fibre and know if they are desired in greater or lesser quantities

- Apply % Daily Values to make healthier food choices
- Recognize popular marketing techniques and misleading front of package claims
- Identify sources of hidden sugars in foods such as fruit juice concentrate, cane sugar, and high-fructose corn syrup

Diabetes Lessons:

Children with type 1 diabetes need strong nutrition literacy to appropriately balance their macronutrients and maintain stable blood glucose levels. Learning how to identify products' carbohydrate and fibre content through modules and recipes supports net carbohydrate calculations and increased accuracy of carbohydrate counting. Furthermore, understanding if a product has significant or insignificant portions of nutrients, such as saturated fats and protein, and knowing alternative terms for sugar commonly used on package ingredient lists helps children determine the nutritional quality and glycemic index of food products and ultimately make healthier choices.

Week 6: Food Waste

Learning objectives:

- Practice environmental stewardship and understand the damage food waste has on the environment
- Differentiate between avoidable and unavoidable food waste
- Discover manageable tips to reduce food waste from daily meals
- Further knowledge of gardening and vegetable growth
- Encourage greater consumption of fruits, vegetables, and plant-based proteins
- Weekly short question quiz to test knowledge

Diabetes Lessons:

Globally, there has been greater recognition of the importance of eating in a more environmentally sustainable manner. In addition to the summerlunch+ modules and recipes which provide children with the skills to prepare nutritious environmentally conscious, plant-focused meals, serving oneself appropriate portions at meals in order to avoid waste is another key lesson. Being mindful of serving sizes and listening to hunger cues prevents leftover food when satiety sets in. This also is a valuable skill for children with diabetes as this will help ensure the dose of insulin administered for the meal is appropriate for the quantity consumed.

Week 7: Healthy Snacking

Learning objectives:

- Understand why processed food should be consumed in moderation
- Discover ways to integrate more nutritious ingredients into favourite meals

- Plan meals and snacks in advance to ensure healthy options are easily accessible
- Learn that juice and sodas contain significant amounts of sugar and whole fruits and drinks including water and milk are nutritious alternatives

Diabetes Lessons:

The learning module will reinforce the importance of substituting and modifying sugar and fat-dense processed snacks with more nutritious alternatives to better manage postprandial response and overall dietary health. When discussing types of snacks, foods will be further categorized as carbohydrate and non-carbohydrate snacks aligning with the terminology used in the SickKids diabetes clinic teachings. Modules will reinforce the importance of selecting foods from both categories as carbohydrates, especially whole grain products, are essential dietary macronutrients. Furthermore, participants will be encouraged to increase the nutritional density and diversity of their snacks by pairing foods from more than one food group.

Week 8: Around the World

Learning objectives:

- Become more open to trying foods with unfamiliar textures, flavours, and scents
- Gain insight into new cultural food practices
- Increase acceptability of peers' cultural lunches and snacks
- Connect deeper to personal culture

Diabetes Lessons:

Eating a diverse diet that supports growth is crucial for children of all health statuses. Exploring new cultural foods expands diet diversity, improves food attitudes, and potentially new sources of nutrition. SickKids serves a diverse population, therefore it is necessary to include cultural foods as part of a healthy diet. Including cultural foods into the diet of each participant requires an individualized approach. The learning module will introduce this concept in a broad sense, and will inform participants to refer to their diabetes management team for more specific dietary information regarding cultural foods.

Supplement 2

Interview Guide

1. Describe your experience with summerlunch+ At Home program.
Probe: Did you enjoy the sessions?

2. Describe your child's experience with summerlunch+At Home.
Probe: Did your child enjoy the session? Did you and your child have enough time to do all the recipes every week?

6. Describe what you and/or your child learned through the summerlunch+ At Home program?

3. Describe your child's ability to access the online program?
Probes: Did your child access particular online tools, website, YouTube videos, google classroom? Describe their experience? Describe any barriers during the program?

4. In your experience, what are the benefits or advantages of the summerlunch+ At Home program?

5. In your experience, what are the challenges or disadvantages of the summerlunch+ At Home program?

7. Did you notice any changes in your child's attitudes towards food and cooking after they participated in the program?

9. Would you recommend the program? If so, why?

11. Did you receive the meal kit or gift card?

12. Meal Kit: Describe your experience with the meal kits?
Probes/followup:
Describe how the food kits affected your participation?
Describe how the food kits affected your satisfaction with the program?
Describe your willingness to participate in this program if the food kits were not provided?

Gift Card: Describe your experience with the grocery card?
Probes/followup:
Describe how the cards affected your participation?
Describe how the cards affected your satisfaction with the program?
Did you have enough time to purchase the ingredients prior to the Saturday sessions?

Describe if you were able to find all the ingredients you needed for the recipes?
Describe your willingness to participate in this program if the grocery cards were not provided?

12. Describe any recommendations for future improvements to the program:
